# Comparison of 24-h and overnight samples of urinary 5-hydroxyindoleacetic acid in patients with intestinal neuroendocrine tumors

**DOI:** 10.1530/EC-12-0077

**Published:** 2013-01-25

**Authors:** Merete Gedde-Dahl, Espen Thiis-Evensen, Andreas Myklebust Tjølsen, Kjerstin Skrede Mordal, Morten Vatn, Deidi S Bergestuen

**Affiliations:** Section of Gastroenterology, Department of Transplantation Medicine Oslo University Hospital Rikshospitale, Postboks 4953, Nydalen0424, Oslo Norway; 1 University of Oslo School of Medicine OsloNorway

## Abstract

Neuroendocrine tumors (NETs) arising in the small intestine are known to produce vasoactive substances, including serotonin, that may result in the carcinoid syndrome (flushing, diarrhea, bronchoconstriction, and carcinoid heart disease). Measurement of the serotonin breakdown product 5-hydroxyindoleacetic acid (5-HIAA) in urine is important in diagnosing and monitoring of patients with intestinal NETs. Our aim was to compare 5-HIAA measurement in 24-h urine sampling with overnight (∼8-h) sampling in patients with known NETs, or at follow-up of patients potentially cured for their NETs. Twenty-four-hour and overnight urine samples were collected from 34 patients and analyzed for urinary 5-HIAA (U5-HIAA) using HPLC. Comparison of the overnight sampling values with the 24-h values showed no difference, *P*=0.45, and there was a significant direct correlation between the two samples using linear regression (*R*=0.97, *P*<0.001). U5-HIAA sample collection during a nightly interval of ∼8 h appears to have the same accuracy as the 24-h collection in this group of patients.

## Introduction

Gastroenteropancreatic neuroendocrine tumors (NETs) are rare tumors of the gastrointestinal tract arising from cells of the neuroendocrine system. These cells are known for their capacity to produce various peptides and hormones with endocrine functions (1). Small intestinal NETs often produce vasoactive substances that may result in the carcinoid syndrome, symptoms of which include flushing, diarrhea, carcinoid heart disease, and bronchospasm. The syndrome is almost exclusively seen in patients with liver metastases, or metastases outside the abdominal cavity, as serotonin and other secretory products from the primary tumor are metabolized in the liver at first pass. Reportedly, 10–18% of patients with small intestinal NETs have carcinoid syndrome (1, 2).

Serotonin (5-hydroxytryptamine) is thought to play a major role in the development of the carcinoid syndrome (1, 3, 4). Biochemically, serotonin is derived from the essential amino acid tryptophan and is primarily found in the gastrointestinal tract (5). In the blood, serotonin is quickly taken up by the platelets (6) and later metabolized, mainly in the liver, in two oxidation steps, resulting in 5-hydroxyindoleacetic acid (5-HIAA) as the main degradation product (5). 5-HIAA is then released into the bloodstream and excreted by the kidneys (7).

Serum measurement of serotonin is possible, but large individual variation makes this measurement unreliable (8). Urinary 5-HIAA (U5-HIAA) has less biological variation and is therefore considered more useful in diagnosis and follow-up of patients with NETs.

In addition to its value in diagnosis and follow-up of patients with NETs (2, 9, 10), U5-HIAA is also associated with prognosis (11, 12). Twenty-four-hour U5-HIAA measurement has a reasonable sensitivity (73%) and up to 100% specificity for NETs (8, 10, 13, 14). The 24-h urine sample collection can, however, be troublesome for both patients and health care professionals and is often performed incorrectly. It requires multiple urine collections during the 24-h period and bulky transport of the sample container(s), with potential social limitations for the patient during the collection. Acidification of the urine is required to maintain stable and sterile conditions in the collection container. Easier methods to monitor U5-HIAA levels are thus warranted.

Zuetenhorst *et al*. (6) investigated the daily variation in U5-HIAA secretion in 4- and 8-h increments and found that the overnight interval (2300–0700 h) was most representative for the 24-h secretion. We therefore wanted to compare the results from an overnight U5-HIAA sampling with results from 24-h U5-HIAA sampling in a well-characterized population of patients, either at work-up of known NETs or at follow-up after surgery with curative intent.

## Materials and methods

### Patients

Ninety-two consecutive patients with histologically verified small intestinal (*n*=89), appendiceal (*n*=2), or proximal colonic (*n*=1) NETs were admitted to the gastroenterology ward at Oslo University Hospital, Rikshospitalet, between September 1, 2006 and September 1, 2007, either for first time evaluation or follow-up after medical or surgical treatment. These patients were included in a study to investigate the relation of carcinoid heart disease to several biomarkers (15, 16), including U5-HIAA. To analyze U5-HIAA in both 24-h and overnight urine samples, the sampling protocol was changed for the last 34 included patients. These 34 consecutive patients were included in this study.

The study was approved by the regional ethics committee, and all participants signed a written consent according to the Helsinki declaration.

### Urinary sampling

All urine samples were obtained during hospitalization and the collection was monitored by nurses trained in the procedure. No food and drug restrictions were given. The sampling started after the first morning's urine was voided and ended by adding next morning's urine to the collection container. Before the next morning's urine was added to the container, a few milliliters were extracted and analyzed separately. During the 24-h collection, the time of the last urination before bedtime and the first morning urine was recorded. The 24-h collections were stored in containers in which 10 ml 2 mol HCl had been added.

### Analysis

All samples where acidified to pH 4 with 6 mol HCl before analyses were performed at the Department of Medical Biochemistry at Oslo University Hospital, Rikshospitalet, using HPLC with electrochemical detection, with a kit from Chromosystems (Munich, Germany). U5-HIAA was measured as μmol/mmol creatinine. The normal range is 0.9–3.8. The lowest detectable value was 0.5 μmol/mmol creatinine.

### Statistical analysis

Differences between groups are given as median and range. Related samples Wilcoxon signed rank test was used to compare the sets of paired U5-HIAA samples. Linear regression analysis was applied to evaluate correlation between the paired samples and between 24-h and overnight collections. As the U5-HIAA values were not normally distributed, the values were plotted using a logarithmic scale. To be able to use all samples in the statistical analyses, samples with values below the detectable threshold of 0.5 were given the value 0.1 μmol/mmol creatinine. Statistical analysis was performed using SPSS 18.0 software (SPSS, Inc.). *P* values are two sided and considered significant when <0.05.

## Results

### Patients

The patient population consisted of 18 men and 16 women, with a median age of 62 (range 18–76) years. Thirty-three patients had small intestinal NETs, while one patient had a NET originating from the appendix. After previous surgical treatment, ten (29%) of the patients were regarded as cured, as no residual tumors were detected on two-phase (arterial and portal venous) computed tomography. Twenty-four (71%) patients had detectable tumors (Table 1). In four patients, both 24-h and overnight sampling was performed twice; hence, a total of 38 paired values were available for the comparison statistical analysis. Median collection time for the overnight sample was 8.3 (range 7–10) h. Collection times were not available for three of the patients.

### Overnight U5-HIAA values compared to 24-h U5-HIAA values

**Figure 1:**
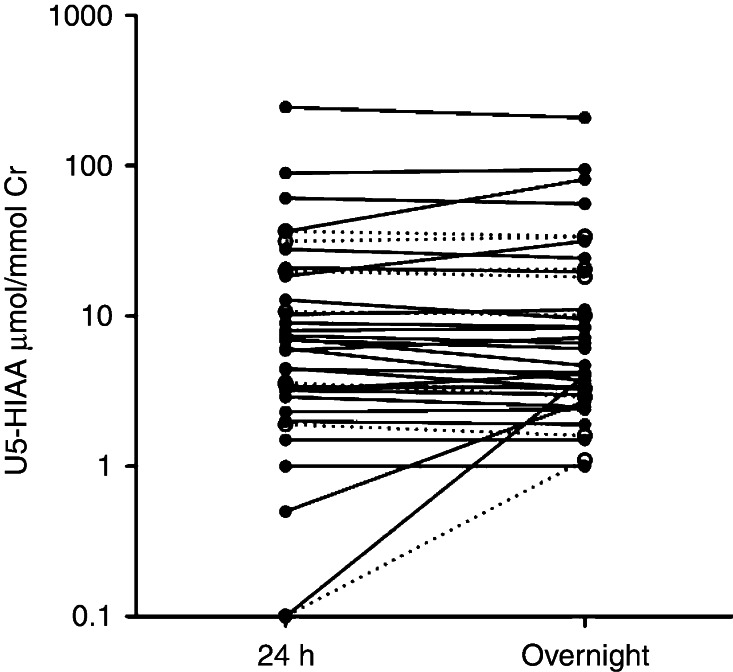
Correlation of 24-h to overnight sampling of U5-HIAA. U5-HIAA values are plotted on the *y*-axis using a logarithmic (log10) scale due to non-normal distribution of the results. The dotted lines represent patients with no detectable tumors and the full lines represent patients with known tumors.

**Figure 2:**
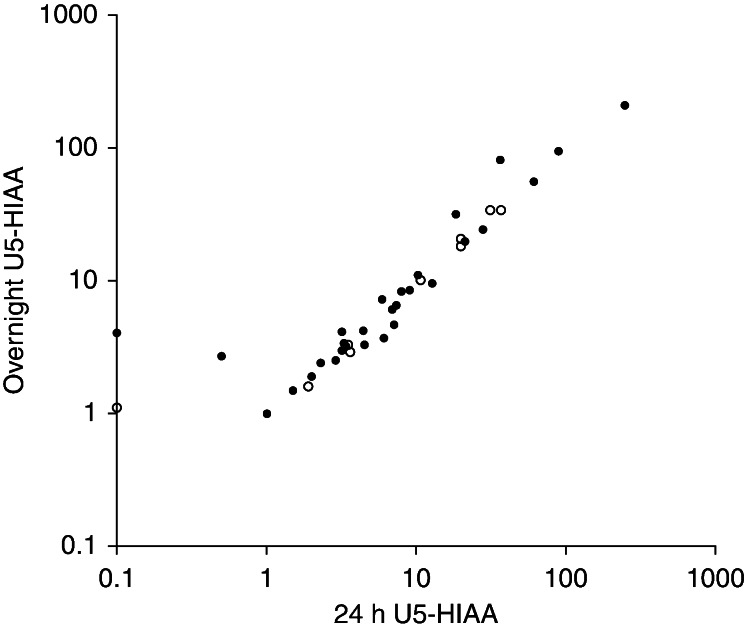
Scatter plot showing the direct correlation between paired 24-h and overnight U5-HIAA samples. U5-HIAA values are plotted on the *x*- and *y*-axes using logarithmic (log10) scales due to non-normal distribution of the results. The open circles represent patients with no detectable tumors and the black circles represent patients with known tumors (*R*=0.97, *P*<0.001).

Measurements of 24-h samples of U5-HIAA (median 6.5 (range 0.1–246) μmol/mmol creatinine) were compared to measurements of overnight samples of U5-HIAA (median 5.4 (1.0–209) μmol/mmol creatinine). No difference was detected between the samples, *P*=0.45. Using linear regression, a significant direct correlation between the 24-h and the overnight sample values for the total group was demonstrated (*R*=0.97, *P*<0.001) (Figs 1 and 2). The patients with no detectable tumors had a median 24-h sampling value of 3.4 (range 0.1–10.7) and an overnight sampling median of 3.1 (range 1.1–10.1). The corresponding results for the patients with known tumors were 8.2 (0.1–89) and 7.8 (1.0–94) respectively. The eight patients using sustained release serotonin analogs (lanreotide or octreotide) had a median of 15.6 (range 1.0–27.9) and 14.6 (range 1.0–31.5) for the 24-h and the overnight samplings.

## Discussion

Our results show a significant correlation between 24-h and overnight U5-HIAA values in this cohort of patients with prior or present NETs of small intestinal or appendiceal origin. This suggests that a U5-HIAA sample collected during a nightly interval of ∼8 h may replace the established 24-h collection for follow-up of these patient groups. An overnight sampling would be less time-consuming and reduce the risk of sampling errors.

This procedure would, however, be susceptible to a diurnal variation in U5-HIAA secretion. Daily variation in U5-HIAA secretion was investigated by Zuetenhorst *et al*. They measured U5-HIAA in 4- and 8-h increments and found that patients fall into two categories, one with little variation in U5-HIAA levels during 24 h and one with more variation. The patients with a pronounced variation had a peak in the morning interval (0700–1100 h) and a dip in the evening interval (1900–2300 h). Overall, they found the overnight interval (2300–0700 h) to be the most representative for the 24-h secretion. The authors speculated whether the variation might be caused by food, drug intake, or activities that influence release of serotonin. Based on their observations, they suggested that overnight urine collection could replace 24-h collection for U5-HIAA analyses (6). In an earlier study, Kema *et al*. (17) found no consistent variations for 5-HIAA in two consecutive 12-h urine samples compared with 24-h samples from 15 healthy adults, which also indicates that a collection interval of <24 h might be sufficient to give a representative picture of serotonin levels.

Still, current guidelines from European Neuroendocrine Tumor Society (18) hold 24-h sampling of U5-HIAA as the standard, and in 2009, they also suggest two consecutive 24-h collections for diagnostic purposes (8). North American Neuroendocrine Tumor Society's consensus guidelines for the diagnosis of NETs 2010 state that 24-h U5-HIAA collection is the most useful measurement for monitoring NETs originating from the intestine (19).

Acidification of the 24-h urine collection sample has been a necessity because of the long storage time. The shorter storage interval required when applying overnight/8-h sampling may very well exclude the need for acidification. However, further research is needed to verify this. Serotonin levels, and consequently U5-HIAA levels, are influenced by certain food and drugs (4). Hence, the European Neuroendocrine Tumor Society Consensus Guidelines recommend that patients follow food and drug restrictions for 3 days before urine collection (8). A limitation of this study is that no food and drug restrictions were given to the patients. However, as any drugs or food items affecting the U5-HIAA levels would most likely influence both the overnight and the 24-h results in the same patient, we do not believe such restrictions would significantly alter the relationship between overnight and 24-h results in this study.

The results from overnight sampling are based on an ‘in body sampling time’ of about 8 h; hence, if patients urinate frequently during the night, all this urine will have to be sampled to maintain a collection interval of ∼8 h.

Somatostatin analog treatment is known to reduce U5-HIAA levels (20). The impact of such treatment on the cyclic variation of 5-HIAA is not known. In our small sample of eight patients using sustained release somatostatin analogs, we saw no difference in the results between the 24-h and the overnight samples. We would not expect any significant variations in U5-HIAA levels as the serum level of sustained release somatostatin analog formulations shows very little daily fluctuation (21). Our results should, however, be reproduced in larger patient groups.

In conclusion, we have demonstrated a significant correlation between paired 24-h and overnight/8-h U5-HIAA samples. Thus, overnight U5-HIAA measurement may replace the more cumbersome 24-h collection in follow-up of patients with intestinal NETs. As our cohort had already been diagnosed with NETs, the accuracy of overnight 5-HIAA measurement for diagnosis of NETs in an unselected population will need further studies. Moreover, our study was performed in a hospital environment and may therefore not be generalizable to outpatient collections.

## Figures and Tables

**Table 1:** Patient characteristics.

	**Total group** (*n*=34)
Gender	
Men	18 (52.9%)
Women	16 (47.1%)
Age, years (median, range)	62 (18–76)
Primary tumor location	
Small intestine	33 (97.1%)
Appendix	1 (2.9%)
Known remaining tumor	24 (71%)
Disease distribution	
Local/regional metastases	5/24 (21%)
Distant metastases^a^	19/24 (79%)
Somatostatin analog use	8 (23.5%)
Overnight sampling period (h)	8.3 (7–10)
Median U5-HIAA, 24 h	6.5 (0.1–246)^b^
Median U5-HIAA, 8 h	5.4 (1.0–209)^b^

a Liver, bone, and lymph glands outside the abdominal cavity (range).

b μmol/mmol creatinine.
